# News and Notes

**Published:** 1900-01

**Authors:** 


### *NEWS AND NOTES.*

---

The Journal-Record had a pleasant call in December from Dr. H. M. Simmons of the *Maryland Medical Journal.*

---

Dr. J. C. Allensworth died in Atlanta on November 30th. He was for many years connected with the National Surgical Institute of this city.

---

Instruction in tropical diseases has been begun at the Johns Hopkins Medical School, no doubt as one result of the commission recently sent to the Philippine Islands.

---

We learn from the *Sei-I-Kwai Medical Journal* that since the new medical-practice law has come into force in Japan about sixty foreigners have applied for a license. Licenses have been granted to fifteen physicians and four dentists, and charters have been granted to seven dispensaries.—*Medical Record.*

---

It is reported that the Czar's skull was trephined at Darmstadt on October 14th, in the hope of relieving certain cerebral symptoms from which he has been suffering. His trouble dates from an assault which was made upon him several years ago when traveling in Japan. It is asserted that the operation was successful, though it is not yet possible to say what the ultimate benefit will be.—*Medical Record.*

---

Fairchild Bros. & Foster recently brought suit against a Chicago apothecary for his having prescribed some other essence of pepsin when their own was designated in the prescription. The court has decided in favor of this well-known chemical firm, and the apothecary will have to pay the costs, amounting to about $500.00. In the future the latter will be restrained from such substitution. We congratulate Fairchild Bros. & Foster.

---

"DOES Practice Pay?" is the title of an editorial in a recent number of the *Maryland Medical Journal.* It states that the practice of medicine is quite uncertain, and that practice, like money, is unevenly distributed. Those wbo have much, gain more; those who have little, lose even what they have. Statistics show that of every one hundred graduates after about twenty years a certain proportion have given up medicine. Some succeed and thrive well; many eke out their existence in a miserable way.

---

THE editor of the *American X-Ray Journal* has written to the dean of every American medical college to find out the name of the doctor who does the X-ray work for that institution. The editor gives the answers in the last issue of the *American X-Ray Journal.* Among them we note the following unique answer from the Georgia College of Eclectic Medicine and Surgery: "In reply to yours of the 2d inst., I will say that we are not doing a great deal in the line you mention. As professor of chemistry I am giving some attention to X-rays."

---

THE practice of medicine in Calabria, Italy, has its drawbacks. In November, in the commune of Cataugaro, a highly respected physician, Dr. Giorgio Vaccaro, was assassinated, and his brother, coming to his relief, was also shot. At Borgia, in the same region, and about the same time, Dr. Pietro Chirac was stabbed by a patient he was attending. The Rome correspondent of the *Lancet,* who gives these facts, remarks that Calabria shows that in the case of a professor considered all the world over as a "sacro-Sanct" against bodily aggression, she can "go one better" than the worst of the semi-civilized or wholly savage of her sister communities.

WE commend the following from the *N. Y. Medical Journal* to the Georgia State Medical Association: "The Medical Society of the County of New York deserves great praise for its assiduity in detecting and punishing offenders against the medical-practice law. According to a report recently furnished to us by the secretary, Dr. William E. Bullard, from May to October, 1895, seventeen prosecutions were brought by the society, with ten convictions; from October, 1895, to October, 1896, fifty-six prosecutions, with thiryt-six convictions; from October, 1896, to October, 1897, fifty-one prosecutions, with thirty convictions; from October, 1897, to October, 1898, thirty-seven prosecutions, with twenty-three convictions; from October, 1898, to October, 1899, forty-five prosecutions, with thirty-three convictions."

---

ACCORDING to *Semaine Med.*, the new regulations in regard to the transportation of persons with infectious diseases in Germany go into effect January 1. A special car must be engaged for persons with smallpox, petechial typhus, diphtheria, scarlet fever, cholera, or lepra. A separate compartment, with water-closet attached, must be engaged by persons with measles, whooping-cough or dysentery. Transportation of persons with the plague is absolutely forbidden. If persons are merely suspected of the above diseases, their transportation will depend on the medical certificate which they may produce. Persons with a visible affection, or who for other reasons are liable to annoy other passengers, need not be transported unless they pay the price of a special compartment, and unless such a compartment can be placed at their disposal on the train.

---

THE following Atlanta physicians were down on the program at the recent meeting of the Southern Surgical and Gynecological Association held in New Orleans December 5–7.

Surgical Shock—Dr. W. F. Westmoreland.

Different Phases of Electric Treatment—Dr. J. McFadden Gaston.

Inflammation of Meckel's Diverticulum Simulating Appendicitis—Dr. Wm. Perrin Nicolson.

Ligation of Lingual Arteries in Operation for Cancer of Tongue—Dr. W. S. Elkin.

Four Interesting Cases of Abdominal Surgery: (a) Pancreatic Cyst; (b) Hepatic Calculi; (c) Fecal Fistulæ—Dr. Floyd W. McRae.

Modification of an Operation for Cystocele—Dr. Geo. H. Noble.

---

IT is customary in Germany for the pupils of a great teacher to express their appreciation and gratitude by dedicating to him a volume of their contributions to learning. The pupils of Dr. William H. Welch, of Baltimore, have decided to give expression to their regard for him in a similar, way and the publication of a volume to mark his twenty-fifth year as a teacher and investigator is now in progress. During the past twenty-five years some seventy-five persons have undertaken investigation under Dr. Welch's leadership, and nearly half of these will contribute to the volume mentioned. The edition will necessarily be limited by the number of subscribers. An early announcement of the publication is made to give opportunity for subscription, so that the committee can decide upon the number of copies to be printed. The volume will be royal octavo in size, and will contain at least five hundred pages of printed matter. It will be illustrated with many lithographic plates and text figures. The price has been fixed at five dollars. The book will contain contributions to pathology and to correlated sciences agreeing in scope with that of the leading scientific journals. All communications and subscriptions should be addressed to Dr. F. P. Mall (Secretary of the Committee of Publication), Johns Hopkins University, Baltimore, Md.

THE following letter is from the president of the American Medical Association and is upon a matter of great importance to the profession:

*To the Members of the Medical Profession in the United States.*

The cause of humanity and of scientific progress is seriously menaced. Senator Gallinger has again introduced into Congress the Bill for the "Further Prevention of Cruelty to Animals in the District of Columbia," which he has so strenuously and misguidedly advocated in the last two congresses. It is Senate Bill No. 34. Twice the Committee on the District of Columbia has, also unfortunately and misguidedly, reported the bill with a favorable consideration. It is speciously drawn to seem as if it were intended only in the interest of prevention to cruelty to animals, but the real object is twofold: 1, to prohibit vivisection, and, 2, to aid the passage of similar bills in all the state legislatures.

It hardly needs to be pointed out that this would seriously interfere with or even absolutely stop the experimental work of the Bureau of Animal Industry and the three medical departments of the government, the Army, the Navy and the Marine-Hospital Service. The animals themselves might well cry out to be saved from their friends. No more humane work can be done than to discover the means of the prevention of diseases which have ravaged our flocks and herds. All those who raise or own animals such as horses, cattle, sheep, pigs, chickens, etc., are vitally interested in the preservation of their health and the prevention of disease.

The inestimable value of these scientific researches as to the prevention and cure of disease among human beings it is superfluous to point out. Modern surgery and the antitoxin treatment of diphtheria alone would justify all the vivisection ever done.

As my attention has been called officially to the introduction of the bill, I take the opportunity of appealing to the entire profession of the country to exert itself to the utmost to defeat this most cruel and inhuman effort to promote human and animal misery and death and to restrict scientific research. It is of the utmost importance that *every physician* who shall read this appeal shall *immediately* communicate especially with the senators from his State, shall also invoke the aid of the representatives from his or other districts in his State, and by vigorous personal efforts shall aid in defeating the bill.

It is especially requested also that all of the national, State and county societies at their next meeting, take action looking toward the same end. If regular meetings are not soon to be held, special meetings should be called. Correspondence is invited from all those who can give any aid.

The committee on the District of Columbia consists of Senator James McMillan, Michigan, Chairman, and Senators J. H. Gallinger, New Hampshire; H. C. Hansborough, North Dakota; R. Redfield Proctor, Vermont; J. C. Pritchard, North Carolina; Lucian Baker, Kansas; C. P. Wetmore, Rhode Island; C. J. Faulkner, West Virginia; Thomas S. Martin, Virginia; Wm. N. Stewart, Nevada; and Richard Kenney, Delaware. Personal letters may be addressed to them or to other senators. Petitions should be addressed to the Senate of the United States.

W. W. KEEN, M.D.,
President American Medical Association.

